# Supplementary material for: All-optical phase conjugation using diffractive wavefront processing
Source: Nat Commun. 2024 Jun 11;15:4989. doi: 10.1038/s41467-024-49304-y (PMC11166986; doi:10.1038/s41467-024-49304-y)
Supplement: Supplementary file 1 — Supplementary Information [file 41467_2024_49304_MOESM1_ESM.pdf]

Supplementary Information for

All-Optical Phase Conjugation Using Diffractive Wavefront Processing

Che-Yung Shen[1,2,3†], Jingxi Li[1,2,3†], Tianyi Gan[1,3], Yuhang Li[1,2,3], Mona Jarrahi[1,3] and Aydogan Ozcan[1,2,3*]

[1]Electrical and Computer Engineering Department, University of California, Los Angeles, CA, 90095, USA

[2]Bioengineering Department, University of California, Los Angeles, CA, 90095, USA

[3]California NanoSystems Institute (CNSI), University of California, Los Angeles, CA, 90095, USA

[†]Contributed equally to the work

[*]Correspondence author: ozcan@ucla.edu

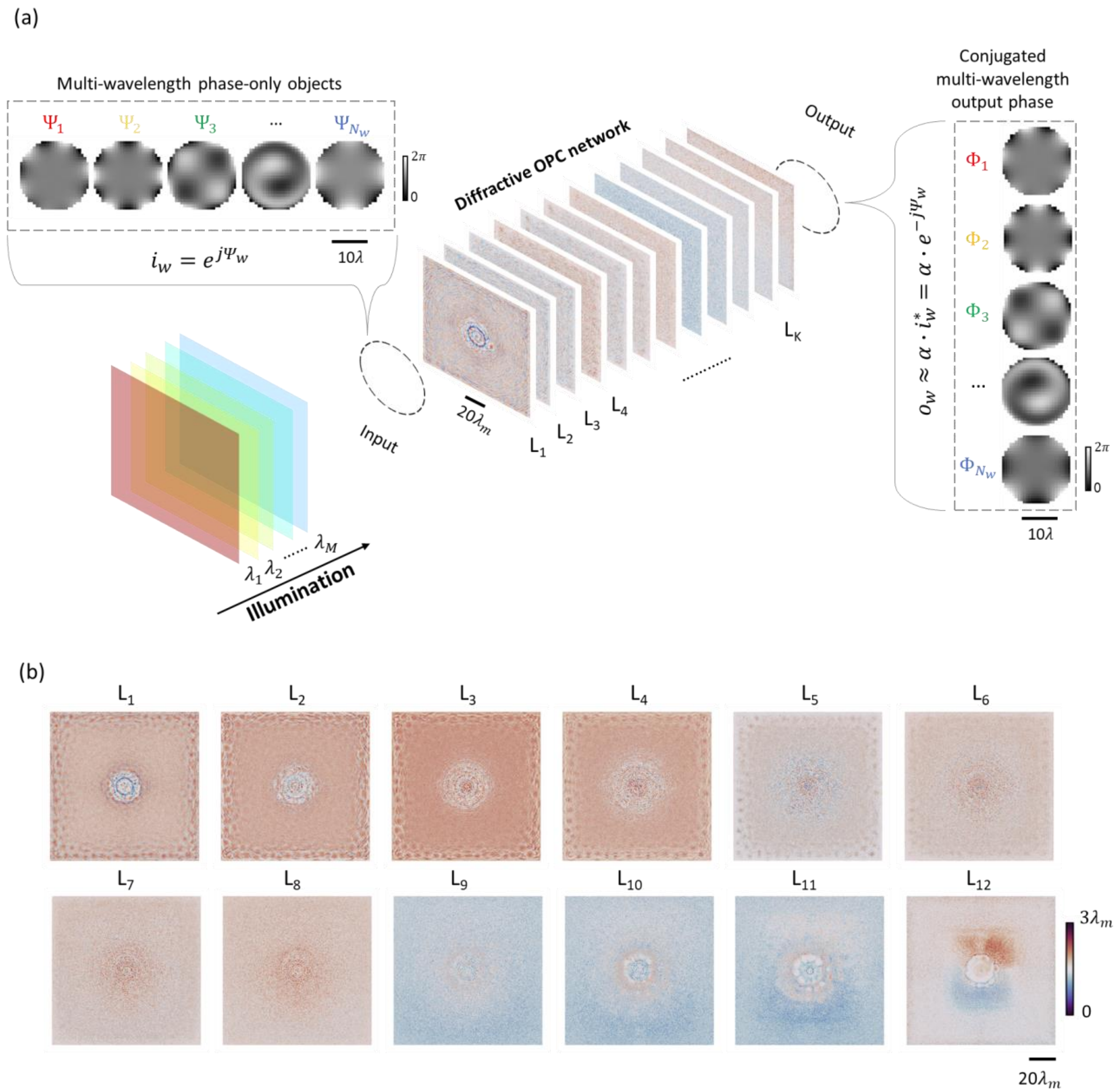

Figure S1: Schematic and operation mechanism of a transmissive diffractive multi-wavelength OPC processor. a, same as Fig. 1a, except performing phase conjugation for N_w distinct wavelengths simultaneously. **b,** Thickness profiles of the resulting diffractive layers for the diffractive multi-wavelength OPC processor trained for N_w = 8.

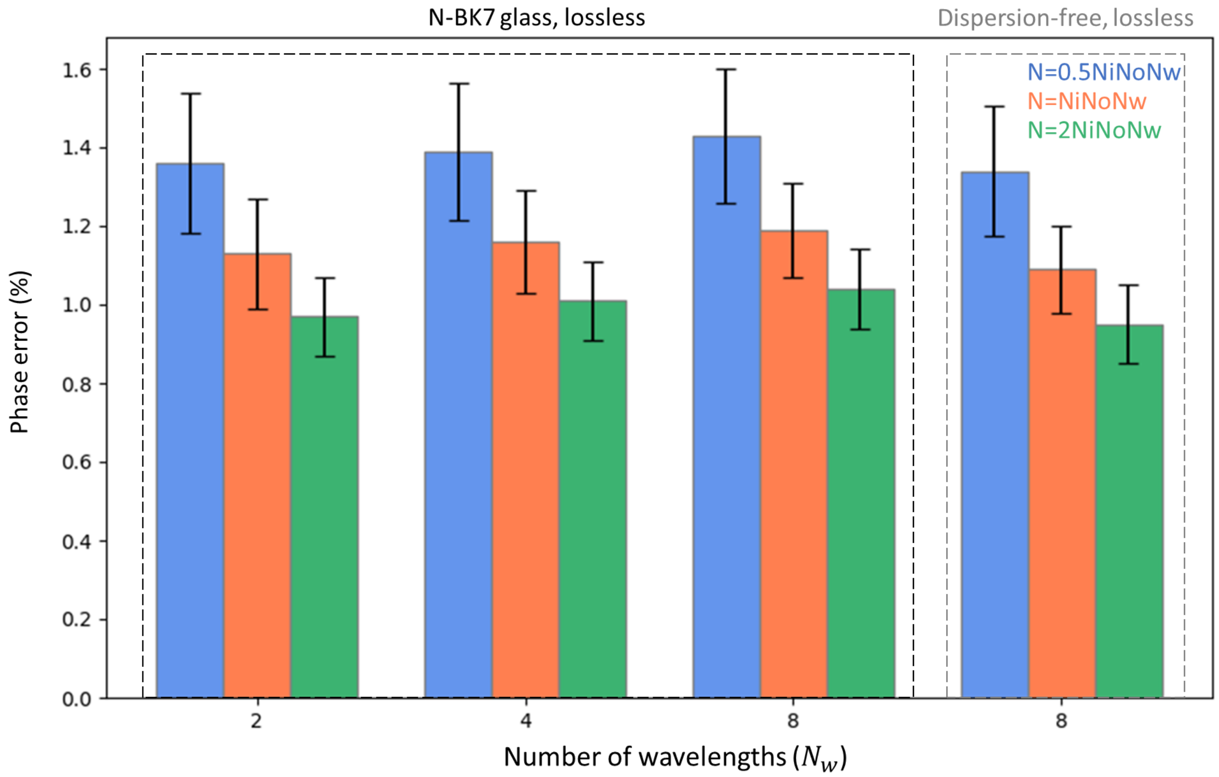

Figure S2: Spectral multiplexing capacity and the scalability analysis of diffractive multi-wavelength OPC processors. Phase error values as a function of the number of wavelengths, N_w. Colored bars represent different numbers of trainable features in the diffractive OPC processor. Metrics are benchmarked across the dataset, reported as mean values with SDs shown as error bars.

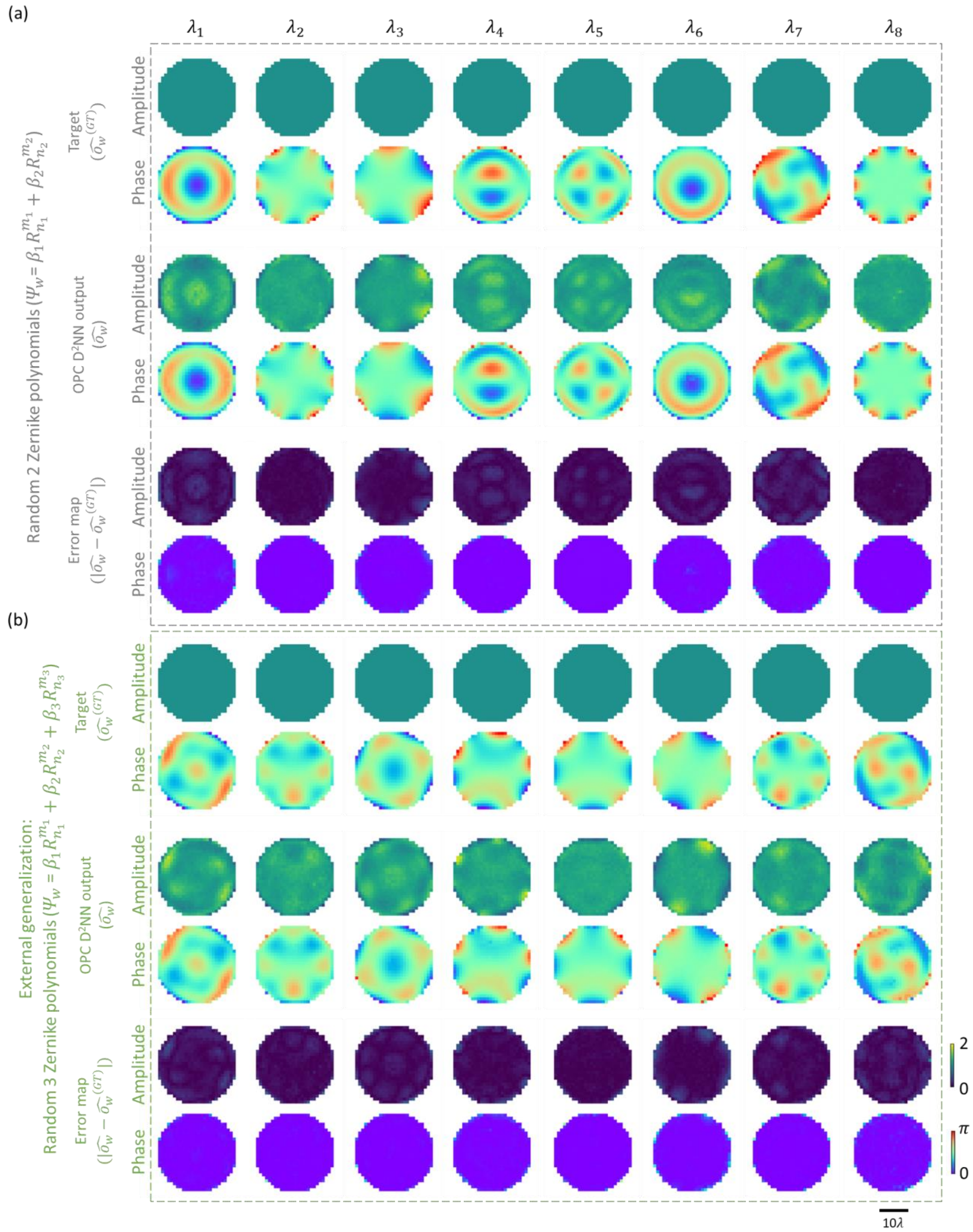

Figure S3: Visualization of the multi-wavelength OPC processor output fields using a broadband diffractive processor operating at the visible band. a, Amplitude and phase profiles of exemplary multi-wavelength output complex fields synthesized by the diffractive

OPC processor fed with phase aberrated input fields; in this case, each wavelength channel has an independent aberration profile, constituted by two random Zernike polynomial terms, never seen during the training stage. For each of these diffractive output fields, its ground truth with perfect phase conjugation is also shown, along with the error map visualizing the absolute amplitude and phase differences between the output field and the ground truth. **b,** Same as (a), except for demonstrating the external generalization capability using three random Zernike polynomials; in this case, each wavelength channel has an independent aberration profile, constituted by three random Zernike polynomial terms, never seen during the training stage. Here, the diffractive multi-wavelength OPC processor performs independent phase conjugation at eight distinct wavelengths $\{\lambda_1, \lambda_2, \ldots, \lambda_8\}$, ranging from 400 nm to 750 nm with 50 nm increments.

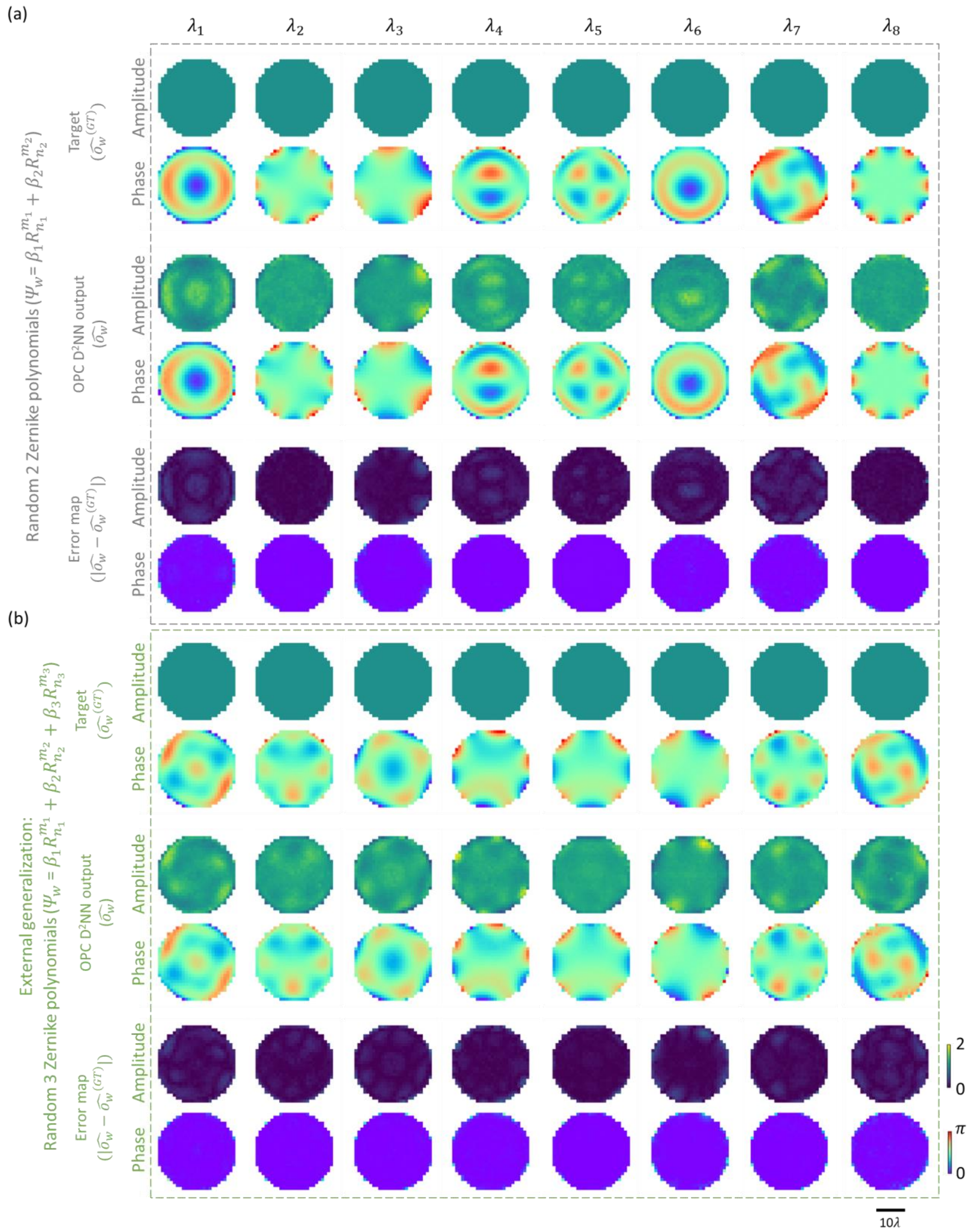

Figure S4: Visualization of the multi-wavelength OPC output fields using a broadband diffractive processor employing a dispersion-free material. Same as Supplementary Fig. S3, except a dispersion-free material was used for the diffractive layers. Here, the diffractive multi-

wavelength OPC processor performs independent phase conjugation at eight distinct wavelengths $\{\lambda_1, \lambda_2, \ldots, \lambda_8\}$, ranging from 400 nm to 750 nm with 50 nm increments.

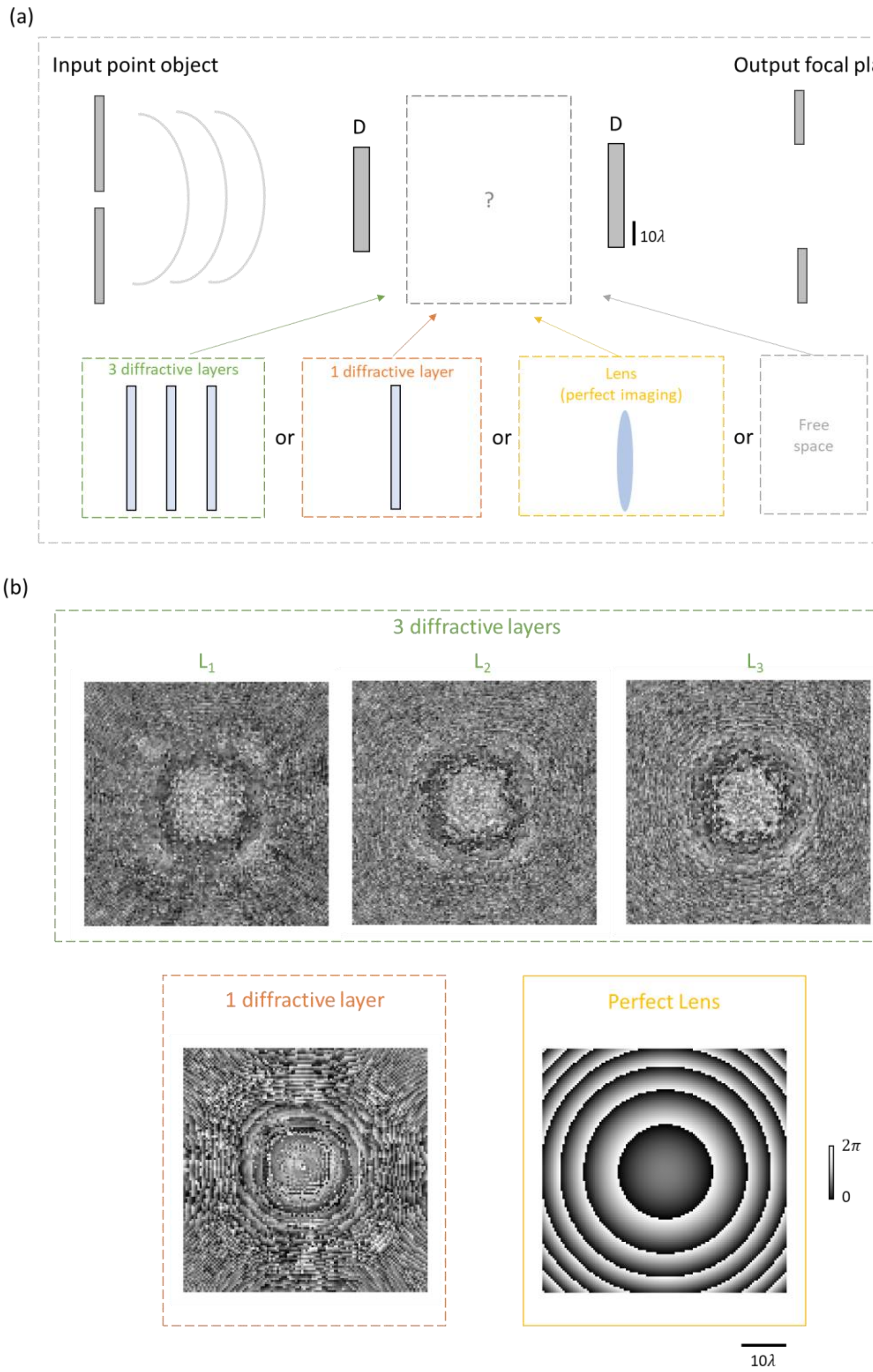

Figure S5. Schematic of alternative designs. a, The same illustration as the one shown in **Fig. 5a** of the main text; four alternative designs are considered here: (1) a three-layer diffractive OPC processor, (2) a single-layer diffractive OPC processor, (3) a thin lens, and (4) free space. **b,** Phase profiles of the trained diffractive layers of the three-layer and one-layer diffractive OPC processor designs, as well as a conventional thin lens.

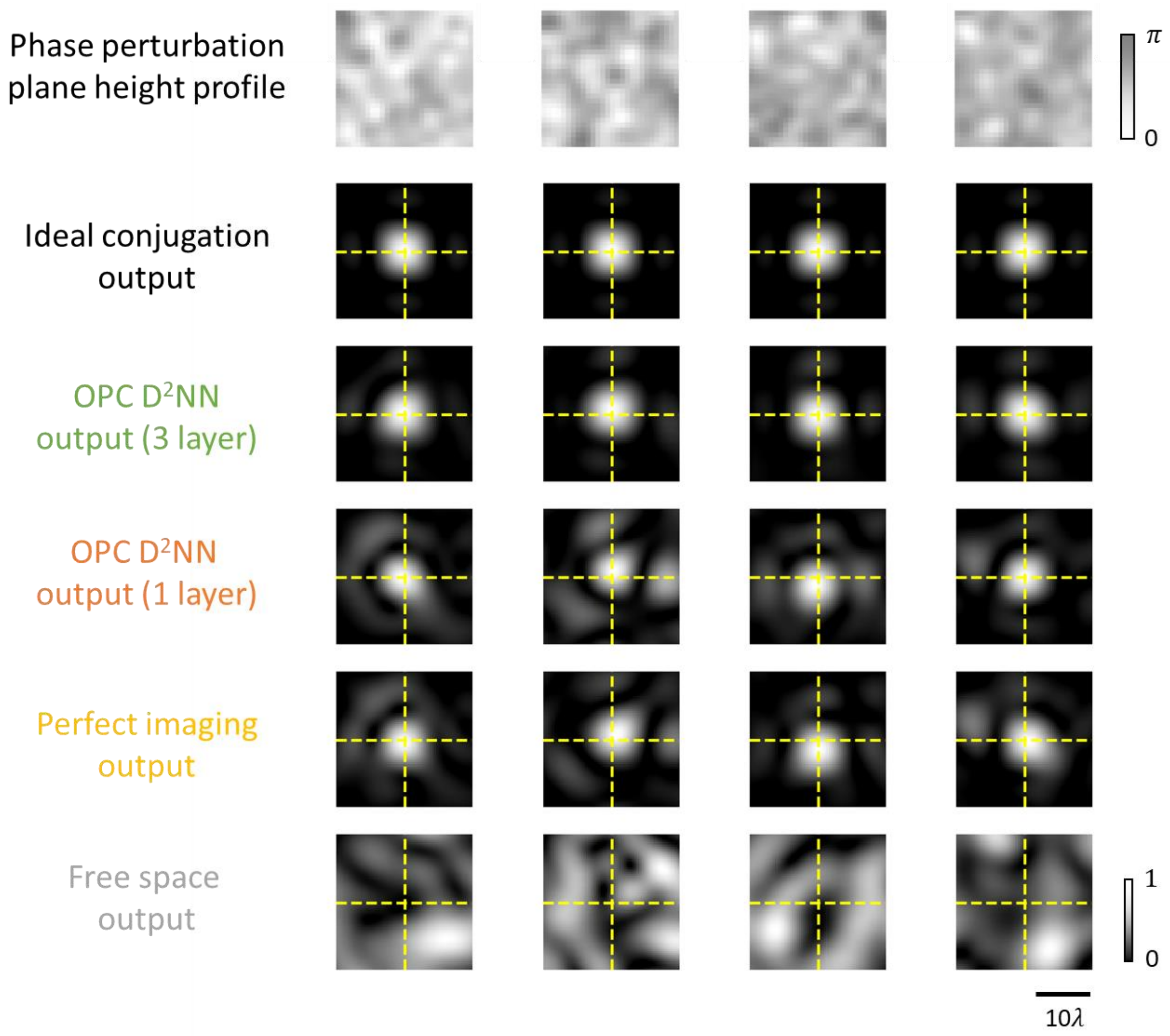

Figure S6. Simulation results for beam focusing through random phase perturbations. The simulated field amplitude distributions at the output plane are shown using the different designs illustrated in Supplementary Fig. S5.

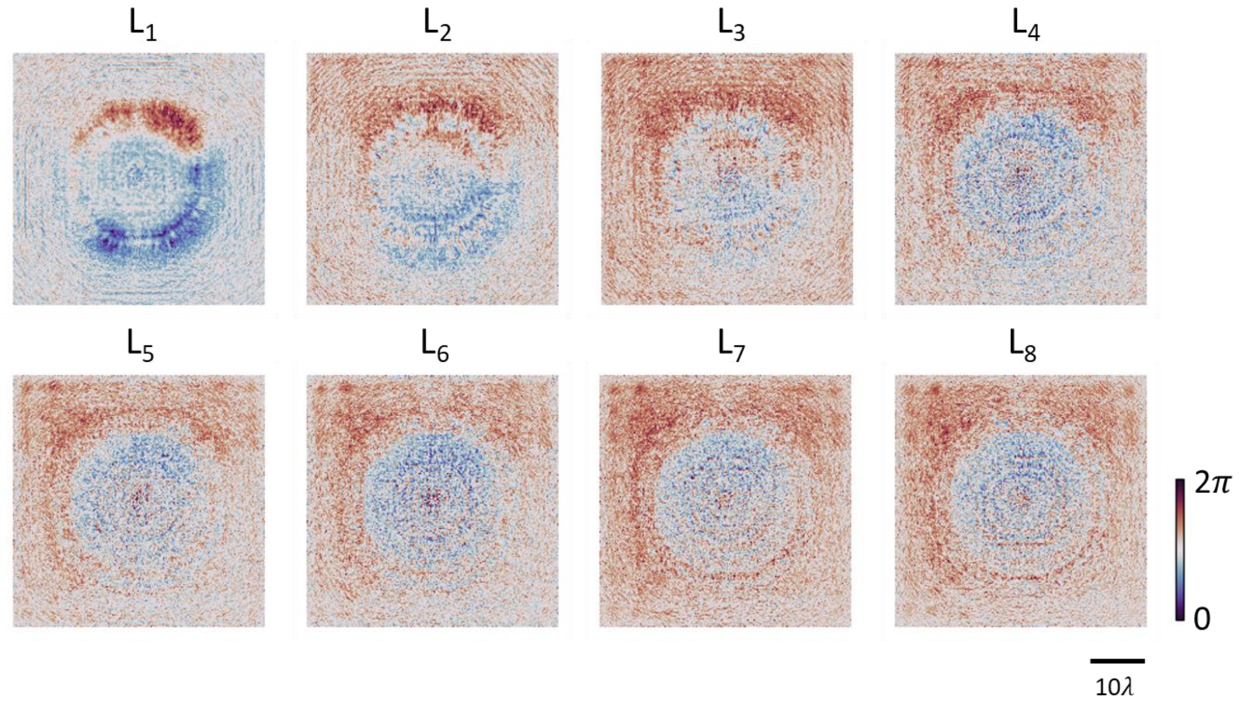

Figure S7. Phase profiles of the resulting layers for the diffractive phase-conjugate mirror design.

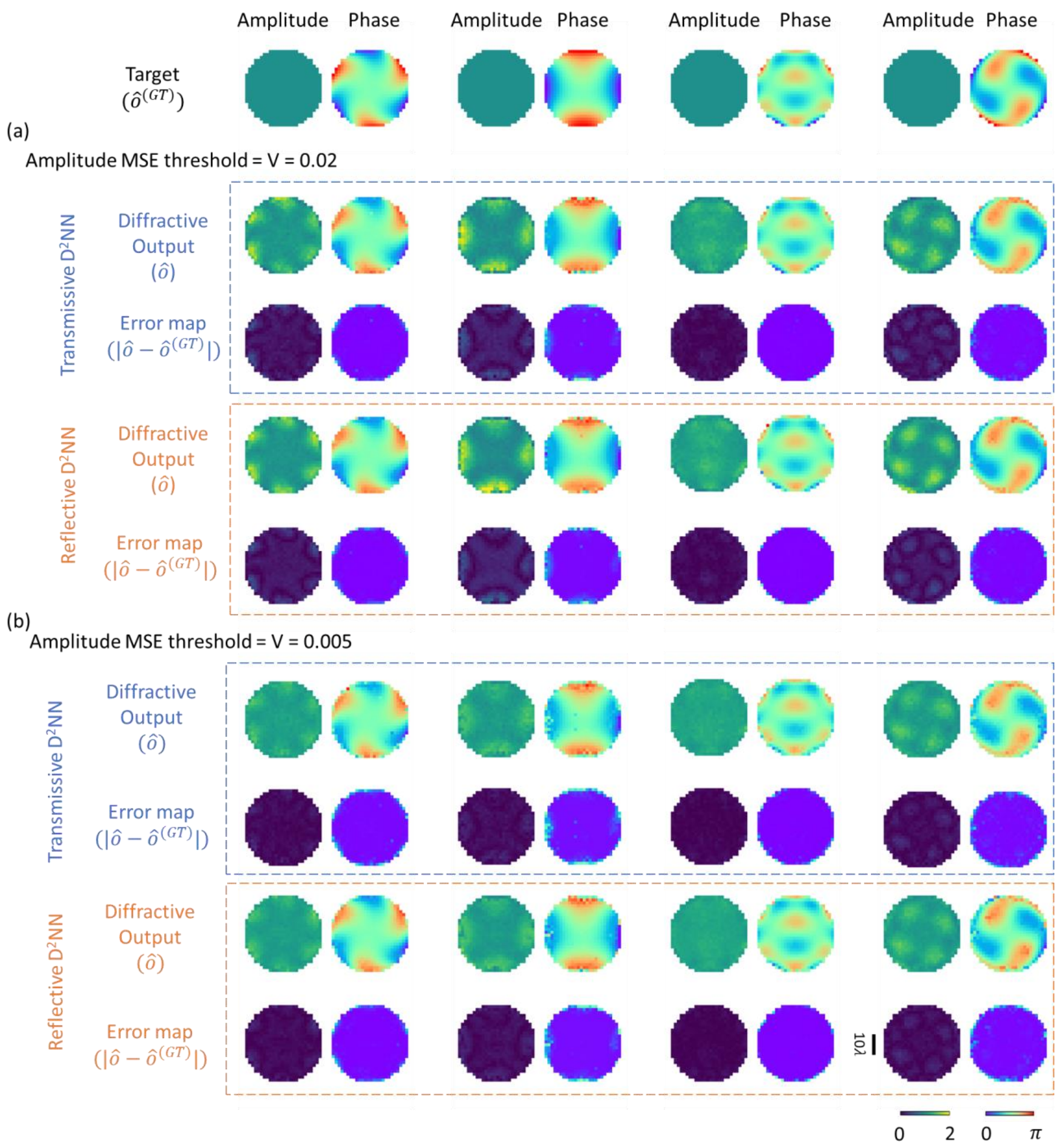

Figure S8. Output visualization of the comparison between the transmissive OPC processor designs and the reflective OPC processor designs using different amplitude MSE thresholds (V) employed during the training stage. a, diffractive output examples of the reflective OPC processor designs and the transmissive OPC processor designs trained with V = 0.02. **b,** same as a., except for V=0.005.

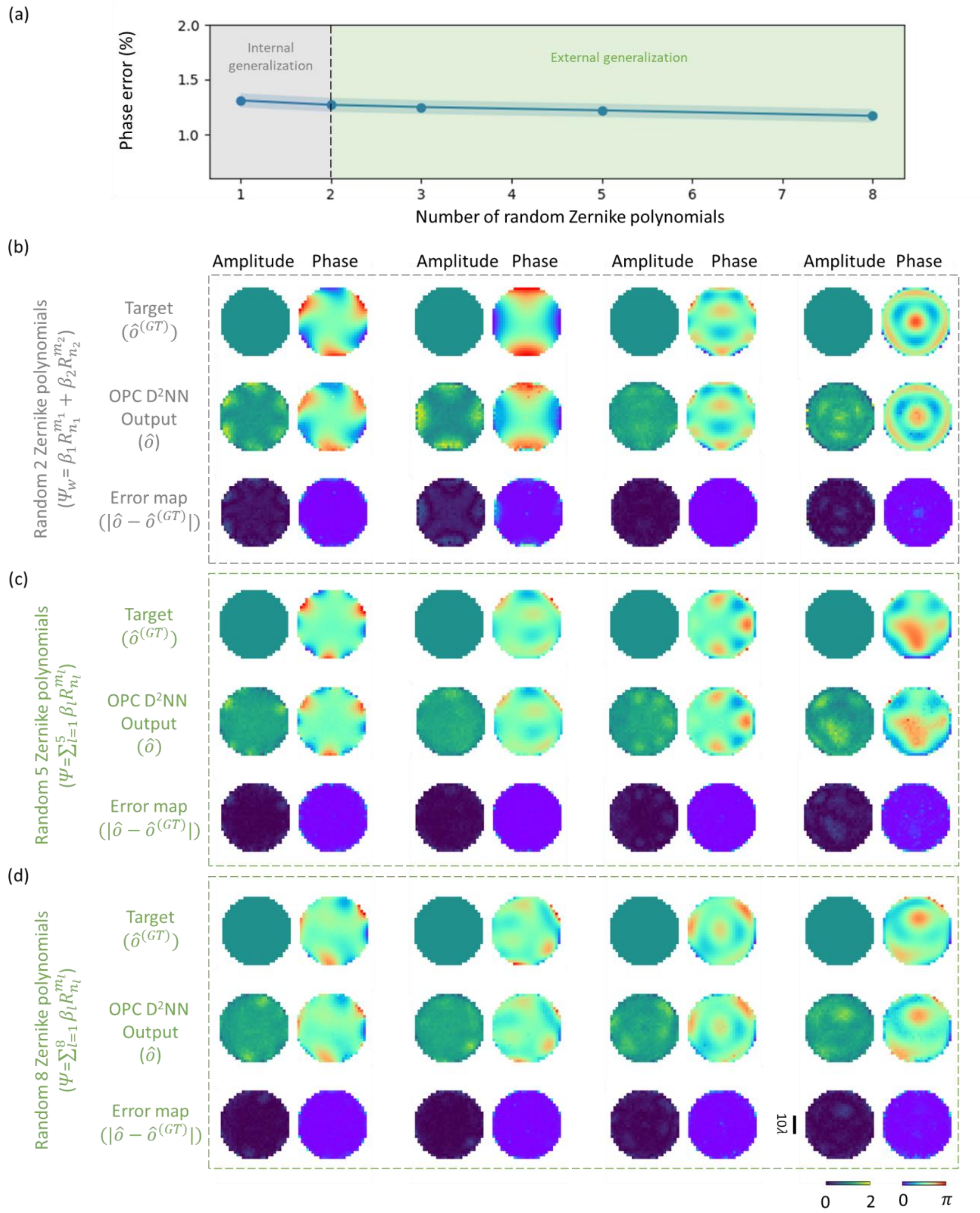

Figure S9. Results for testing the external generalization performance of the diffractive OPC processor design. a, Phase error values of the diffractive OPC processor outputs as a function of the number of Zernike polynomials. The blue transparent area indicates the range of the standard deviations. **b,** same as **Fig. 2c** (main text). **c,** same as (b), but the aberrated input

fields are constituted by five randomly selected Zernike polynomial terms, never seen during the training stage. **d,** same as (b), but the aberrated input fields are constituted by eight randomly selected Zernike polynomial terms, never seen during the training stage.

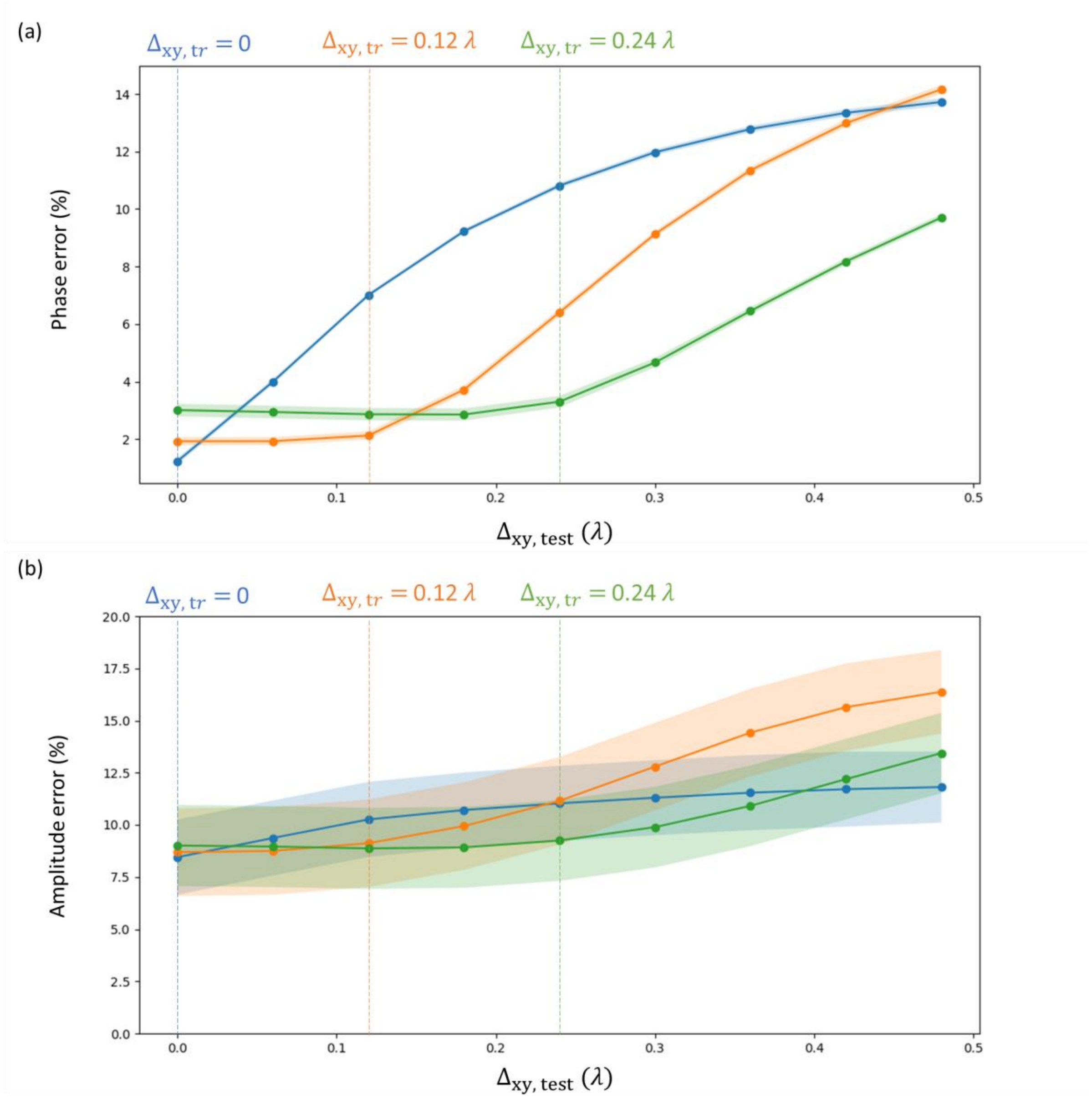

Figure S10. The impact of lateral misalignments on the phase conjugation performance of the vaccinated diffractive OPC processors. a, Phase error values of the diffractive OPC processor outputs as a function of random lateral shifts. **b,** Amplitude error as a function of random lateral shifts. In both plots, transparent areas indicate the range of the standard deviations. The blue curves represent the performance of the diffractive OPC processor previously shown in **Fig. 2a;** the other curves (green and orange) represent the vaccinated diffractive OPC processor designs. Specifically, when the diffractive OPC processors were trained with random lateral displacements of the diffractive layers, the corresponding blind testing phase error maintained a low level of <4% when the random misalignment used in the testing phase did not exceed the maximum misalignment magnitude used in the training phase.

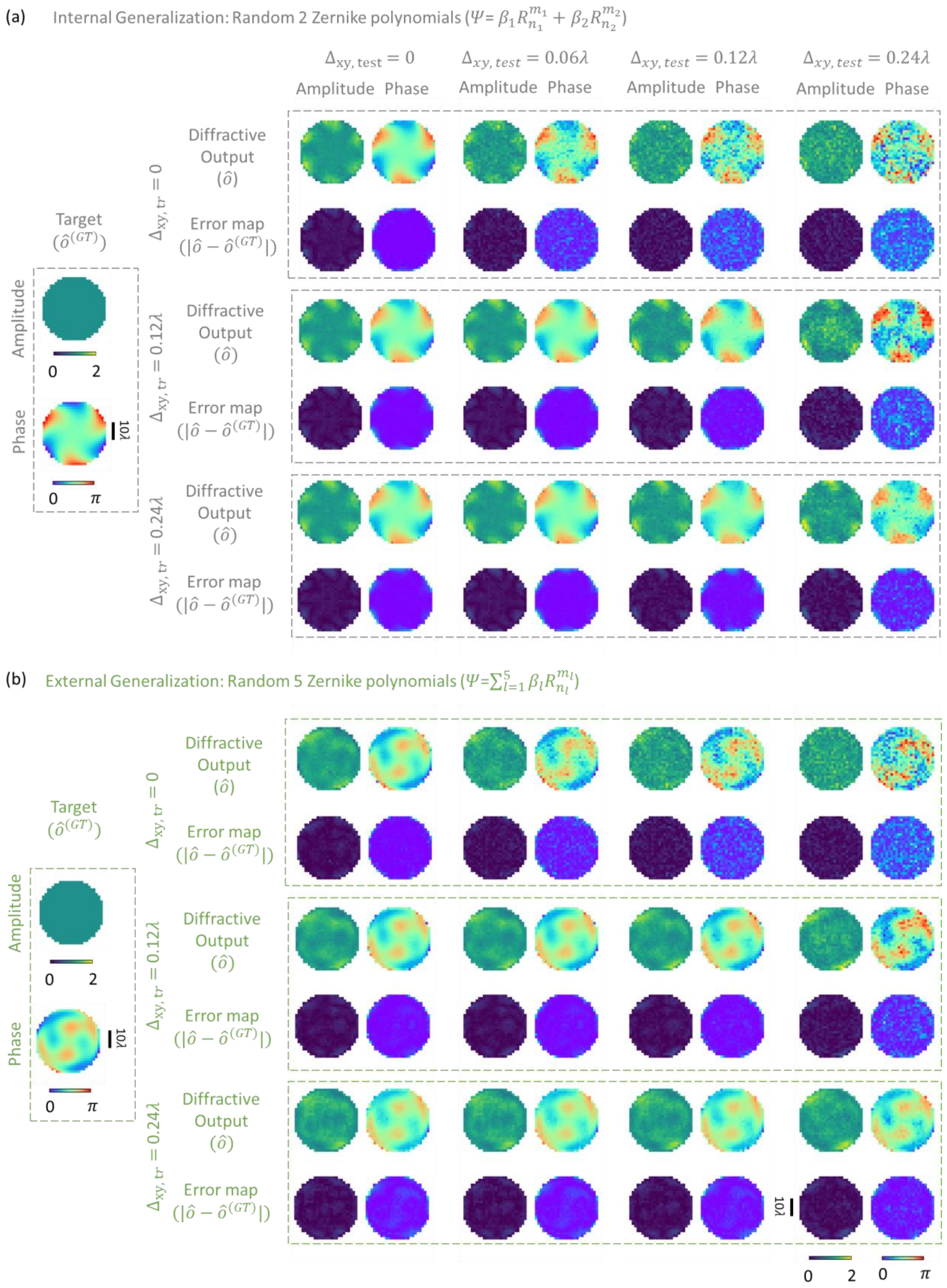

Figure S11. Output visualization of vaccinated diffractive OPC processors with different

degrees of lateral misalignments. Examples of the diffractive OPC processor output fields with different degrees of vaccination. **a,** The aberrated input fields are generated by the combination of two randomly selected Zernike polynomials. **b,** same as (a), except that five randomly selected Zernike polynomials were used.

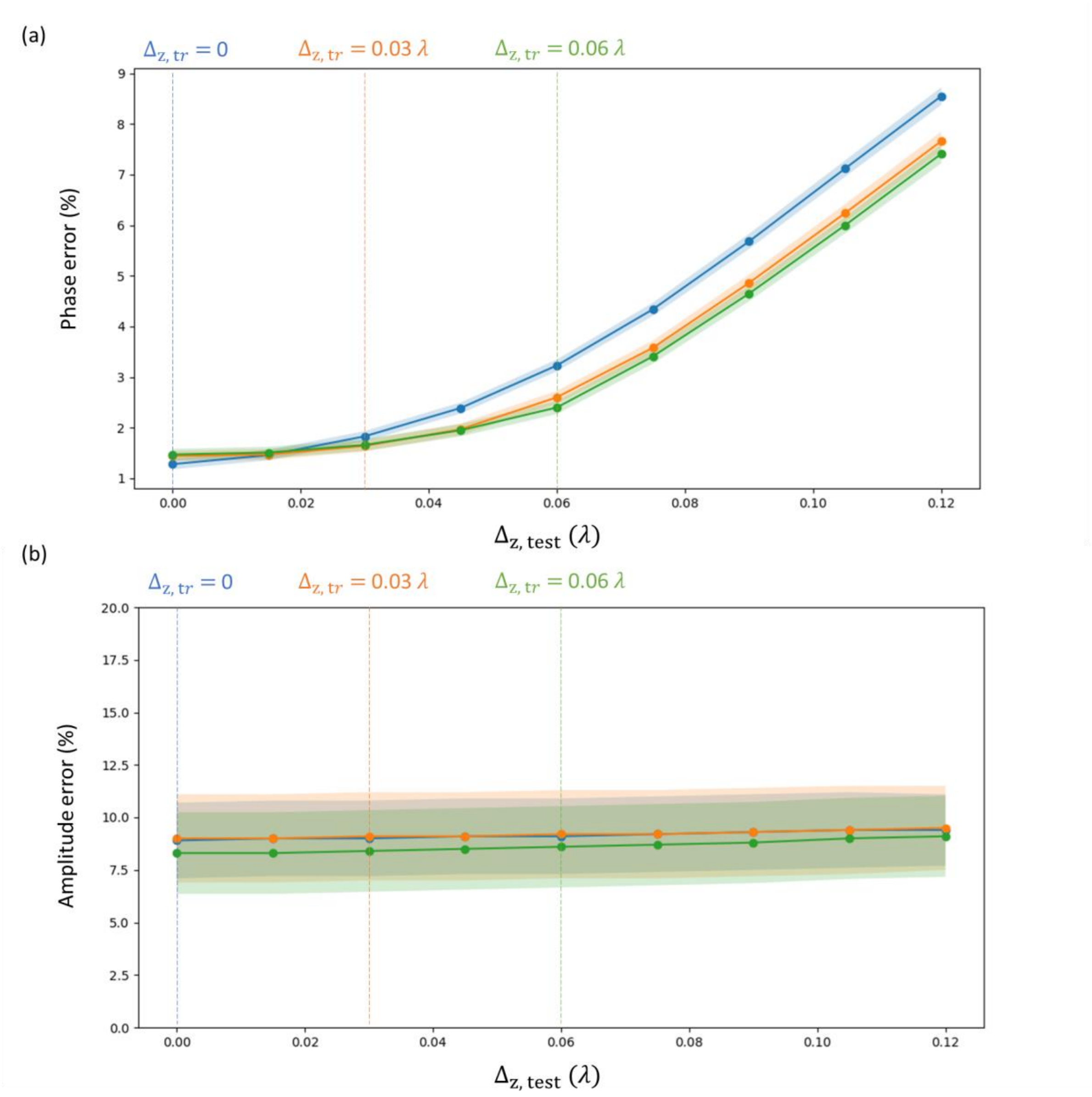

Figure S12. The impact of axial misalignments on the phase conjugation performance of vaccinated diffractive OPC processors. a, Phase error values of the diffractive OPC processor outputs as a function of random axial shifts. **b,** Amplitude error values of the diffractive OPC processor outputs as a function of random axial shifts. In both plots, transparent areas indicate the ranges of the standard deviations.

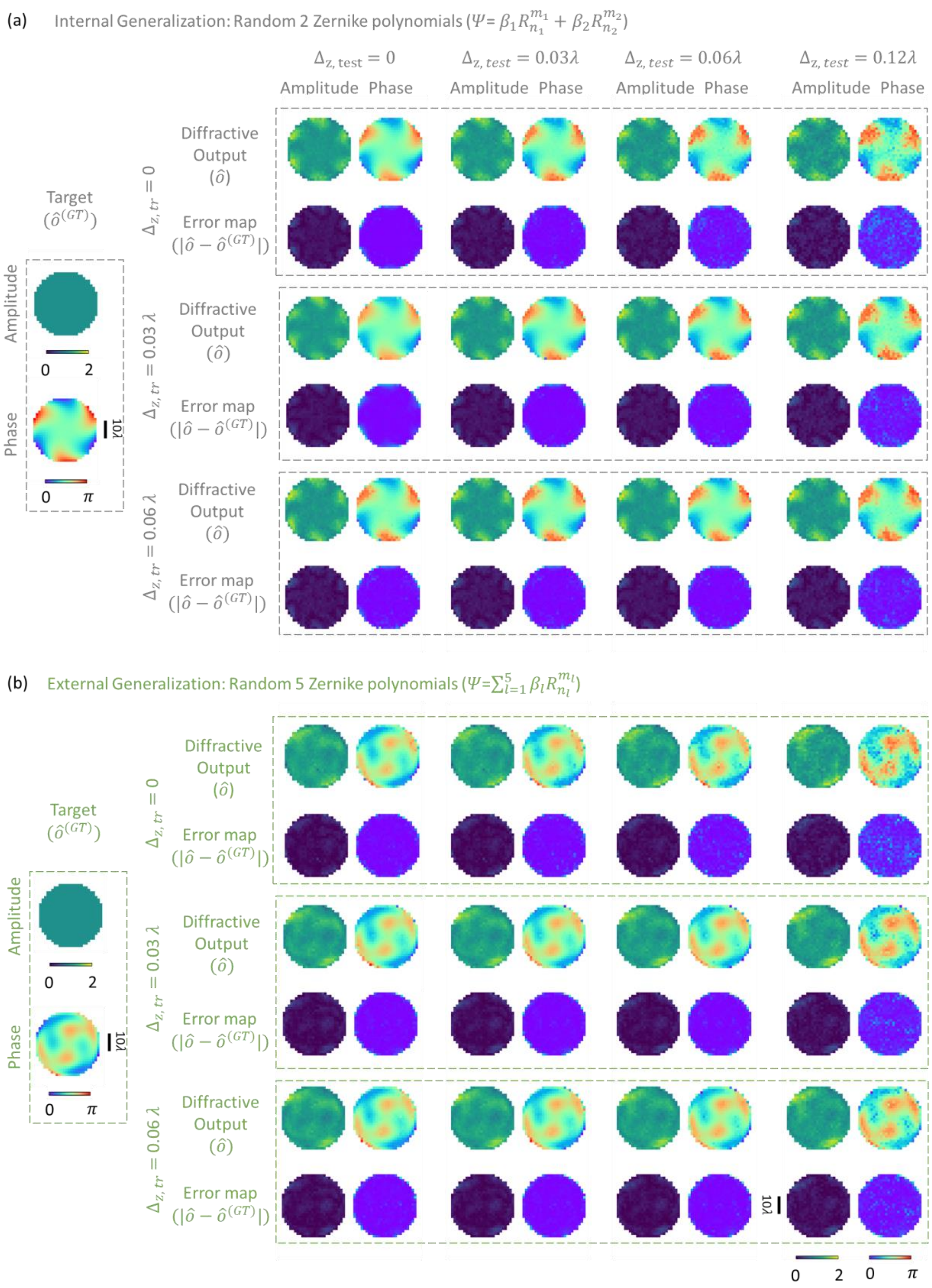

Figure S13. Output visualization of vaccinated diffractive OPC processors with different degrees of axial misalignments. Examples of the diffractive OPC processor output fields with

different degrees of vaccination. **a,** The aberrated input fields are generated by the combination of two randomly selected Zernike polynomials. **b,** same as (a), except that five randomly selected Zernike polynomials were used.
